# From isolation to revival: trade recovery amid global health crises

**DOI:** 10.1186/s12992-024-01048-6

**Published:** 2024-05-06

**Authors:** Lijuan Yang

**Affiliations:** School of Economics, Lanzhou University, Lanzhou, 730000 China

**Keywords:** Global health events, National trade recovery, Trade normalization, Measure design, Multi-case comparative analysis, F02, F10, F14, L63

## Abstract

**Background:**

The COVID-19 pandemic has highlighted the importance of designing effective trade recovery measures in response to global health events (GHEs). This study combines international trade risk management theory and multi-case comparative analysis of past GHEs to present a theoretical framework for designing national trade recovery measures for future events.

**Results:**

The research finds that during GHEs, trade risks shift to fundamental uncertainty, requiring spatial–temporal-subject dimension recovery measures. The study suggests changing the focus of trade recovery policy design from emergency-oriented and single-dimension measures to reserve-oriented and enduring-effect measures of comprehensive dimensions at micro- and macroeconomic levels.

**Conclusion:**

The study contributes to the debate on managing trade risks in times of crisis, where there is a need to develop effective trade recovery measures that account for the complexities of global trade and the unique challenges of GHEs. The findings provide practical guidance for trade officials and policymakers to design measures in response to GHEs to improve a country’s overall trade recovery.

## Background

Global health events (GHEs), defined as pandemics or crises that widely influence people’s health, have major repercussions. Countries affected by GHEs^1^ need to implement trade recovery measures to resume trade [1].^2^ These measures are crucial for mitigating the risks of capital, trade, and supply chain disruptions caused by disasters, reducing the burden of epidemics, and boosting national and global economies. Current research on GHEs is concentrated on medicine and public health issues, and only a few economic studies have been conducted [2–5]. Even this limited research has tended to peak alongside health events and bottom out when they end. The process of recovering from GHEs by taking comprehensive measures has rarely been discussed. Therefore, by applying international trade risk management theory and multi-case studies, this study examines the design of national trade recovery measures and offers countermeasures for GHEs.

Fifteen GHEs have occurred since the 1990s (Table 1), highlighting that their economic impact exceeds their immediate health consequences and regional spread [6]. Geographically distant health events can potentially reverberate to unaffected countries through international trade [7]. GHEs adversely affect the country of origin, trade partners, and the global economy [8]. Globalization has further exacerbated this negative impact.^3^ GHEs affect foreign trade.^4^ The transfer effects of trade bans can drastically harm welfare [9, 10], leading to structural fractures in imports and exports [11]. Moreover, biosecurity measures during disease outbreaks [12] can indirectly influence technical trade measures that affect emerging countries’ exports to developed countries.

**Table 1:** GHEs since the 1990s

Year	GHE	Affected Areas
1994	Plague	India
2003	Severe acute respiratory syndrome (SARS)	China
2004	Avian Influenza H5N1	Asia
2009	Influenza H1N1^a^	Mexico, Canada, United States
2011	Nuclear leakage	Japan
2012	Middle East Respiratory Syndrome (MERS)	Middle East
2014	Poliomyelitis	Central Asia, Middle East, Central Africa
	Ebola^a^	Guinea, Sierra Leone, Liberia
2015	MERS	Korea
	Zika	Brazil
2016	Zika^a^	Maldives, New Caledonia
2017	Measles	Europe
2018	Ebola^a^	Congo
2019	Dengue	Latin America and Asia
2020	Coronavirus disease 2019 (COVID-19)^a^	Global

^a^Health events announced by the World Health Organization (WHO), post-establishment of international health regulations. Source: Authors’ overview

Scholars advocate the following strategies to respond to GHEs. (1) Conducting response measures. Once a global health emergency is under control, it enters the international trade recovery stage. The countries involved in the event must renegotiate trade agreements with their trading partners, strengthen consumer expectations and confidence, and evaluate response measures [13, 14]. (2) Planning and sequencing measures. The international trade recovery must transcend the risk model to plan and prioritize trade recovery measures based on the interdependence between public health and trade [15, 16]. The affected areas must take pre- and post-prevention and mitigation measures after the disaster outbreak [17]. (3) Implementing regional measures. The United States (US) adopted regional measures to manage the highly pathogenic avian influenza pandemic and to resume and maintain trade. Trading partner countries accepted the regional recommendations and allowed poultry and poultry product imports from US regions without the disease [18].

Furthermore, countries must address factors influencing trade recovery, as various factors determine the extent of adverse effects on trade and the duration of recovery. Emerging countries need to follow and strictly enforce the standards of the World Health Organization (WHO) [19] and deal with dynamic changes in trade and supply chain nodes during GHEs [20–24]. These efforts should include modeling and scenario simulation based on epidemiology and economic theory [25, 26], risk rating of GHEs [27], and artificial intelligence modeling for potential risks [28]. The models under continuous development must reflect the dynamic landscape of emergent situations [29].

Although the WHO and multilateral institutions do not recommend interrupting international trade following a GHE, limited research has provided targeted suggestions for countries to adopt an appropriate course of action [30, 31]. The interconnectedness of global health and the global economy highlights the need for such a policy and the relevance of health security efforts [7] to mitigate immediate health risks and long-term economic disruption. Including economic policies as part of GHE policies leads to collaboration between epidemiologists and economists in an economy-wide pandemic or public health crisis modeling, thereby demonstrating potential benefits [32, 33].

This study posits that trade recovery is a dynamic process; hence, designing appropriate trade recovery measures should consider spatiotemporal dimensions and specific stakeholders at various subject levels. Research on developing trade recovery measures covers the spatial dimension but overlooks the time dimension. Lee et al. [34] established spatiotemporal modeling but did not distinguish different subject levels. Combining the spatiotemporal dimension and specific subject levels in trade recovery measures is essential for their success, ensuring adaptability and coverage across diverse economies. Additionally, comparative studies on countries’ trade recovery after different GHEs are limited, and research on trade recovery measures remains restricted to a single dimension. A clear framework for countermeasures is yet to be developed.

## Methods

This study investigates trade recovery measures in countries affected by GHEs. The method includes a theoretical analysis based on international trade risk management with comparative multi-case studies. This methodology was developed by scholars such as Stake [35] and Yin [36], who formalized the approach as a tool for conducting in-depth explorations within real-life contexts. This qualitative research method enables the examination of complex phenomena within their specific settings, making it particularly suitable for understanding the nuanced implications of trade recovery measures across different geopolitical and socio-economic landscapes.

We construct a time–space-subject recovery measure framework, combining cases from the trade recovery measures adopted by Mexico, the US following the outbreak of influenza A H1N1, Japan following nuclear leakage triggered by a tsunami, three West African countries (Guinea, Liberia, and Sierra Leone) following the outbreak of Ebola, and South Korea following the outbreak of the Middle East Respiratory Syndrome (MERS). The framework is to design trade recovery measures for possible future events and for countries that are yet to recover from the COVID-19 pandemic.

Historically, the case method has been leveraged in public health and international policy research [6, 12, 37], offering insights into policy development and implementation. Its adoption in this study, rather than more quantitative methods, allows for a deep, contextual analysis of policy effectiveness and adaptability in diverse scenarios, thereby enhancing our understanding of trade recovery strategies. The application of this framework also supports the synthesis of cross-sector policies, combining health imperatives with economic resilience to devise trade recovery roadmaps for both immediate and long-term strategic planning.

This study’s theoretical and practical contributions are as follows. (1) Exploration of spatiotemporal dimensions and subject-specific levels enriches the design of trade recovery measures, expanding extant research in international trade risk management theory by integrating contextual analysis into risk assessment and mitigation strategies. (2) A comparative analysis of consistency and heterogeneity in trade recovery measures across developed and emerging countries reveals gaps in public health emergency response mechanisms related to international trade, deepening the need for tailored strategies targeting specific economic vulnerabilities. (3) Insights obtained offer references for shaping national trade recovery policies in response to GHEs. Given the post-disaster recovery’s uncertainty, governments must enforce transformative measures [37], which are both adaptive and robust, to ensure economic stability and resilience.

## Results

### Theoretical framework for designing trade recovery measures in GHE-affected countries

According to the international trade risk management theory [14, 38], the health event emergency management system includes four stages: early warning, preventing spread, controlling or eliminating the event’s impact, and recovery. The emergency’s containment initiates the recovery phase. Countries and regions have a low proportion of recovery work in the health emergency management system, which must be fully developed for trade recovery from GHEs [14]. During the international trade recovery stage, the affected country must renegotiate trade agreements with its partners, enhance expectations, disseminate information to consumers, and evaluate the implementation effect of the trade recovery measures. Promptly identifying international trade risks and employing risk management measures can prevent and mitigate risks and ensure the smooth progression of trade.

During GHEs, trade policy, market demand, and competition vary; exchange rates between a country and its main trading partners fluctuate; or fixed rates are maintained at a significant cost, leading to objective risks. Although trade subjects cannot eliminate objective risks, they can actively prevent them. GHEs expand the scope of restrictions on the movement of people and goods, with continuously increasing uncertainty within the affected country’s trade environment. The subjective risk of decision-making errors and improper measures increases sharply as governments, organizations, and people face multiple pressures [39] and emergent behaviors.

GHE-initiated international trade risks are multidimensional, featuring a spatiotemporal evolution. Measured in time, an epidemic’s early, middle, and late stages face short- and medium-term risks. The risk extends from the epidemic’s origin to neighboring countries and major trading partners. With aggravating uncertainties and risk factors, the potential impact of GHEs on trade expands beyond short-term scales and localities, further increasing the complexity of trade recovery.

Subject levels, including international, national, industrial, enterprise, and consumer, simultaneously face systematic risks caused by GHEs. The cognitive prediction of events leads to pressure superposition, unbalanced considerations, and decision-making errors, thereby increasing the risk of improper measures. These risks are intertwined throughout GHEs, making it more difficult for affected countries to recover their normal trade levels [14–16]. Recovering from health events through only one type of measure is infeasible.

**Fig. 1:**
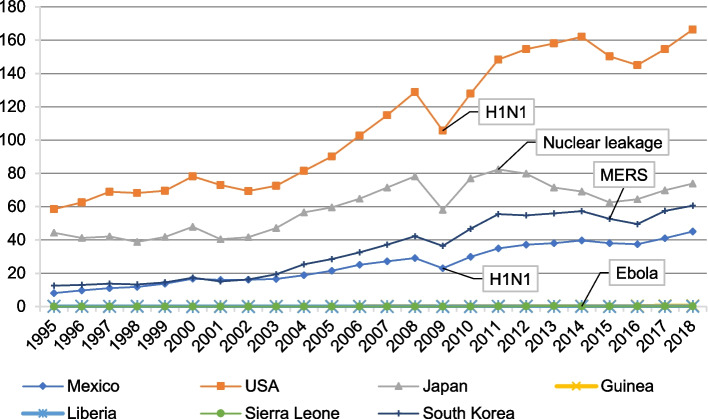
Trend of goods exports in countries with GHEs, 1995–2018 (current price, USD 10 billion)*.* Source: Author’s analysis based on data from the World Bank Database

To address the international trade risks triggered by GHEs, the trade recovery countermeasures of affected countries must be strengthened in their spatiotemporal dimensions and include international, national, industrial, enterprise, and consumer groups for different subject levels. Efforts should include tracking the epidemic’s evolutionary stage and identifying its regional characteristics, as shown in [18, 40], which highlighted the effectiveness of region-specific trade policies during the Ebola outbreak. Moreover, it is necessary establish a national trade recovery countermeasure repository featuring adequacy, flexibility, and completeness. These measures are essential to shift from an emergency single-trade recovery measure design to a comprehensive, long-term trade recovery measure design (Fig. 1).

### Comparative case study on trade recovery measures after GHEs

This study comparatively analyzed the trade recovery measures of relevant countries in the aftermath of four GHEs: the H1N1 influenza that developed in Mexico in 2009 and spread to the US, affecting both countries and their major trading partners; the 2011 Fukushima nuclear leak; the 2014 Ebola virus outbreak that spread rapidly in West Africa; and the 2015 MERS outbreak introduced to South Korea by international travelers. Despite their sudden onset, these GHEs triggered various national trade recovery measures because of differences in their nature.

A comparative case study methodology, conducive to exploring the characteristics of national trade recovery measures and the specifics of the events [41], supported by Yin [36] for its effective analysis of complex phenomena within realities, was applied. This method involves a systematic collection, comparison, and analysis of case data to identify patterns, test theories, and derive insights that are not apparent through singular case analyses. In implementing this methodology, this study meticulously documented the sequence of trade policy adjustments, timing (spatiotemporal dimensions), and targeted entities (subject dimensions) for each GHE case. This approach enabled the identification of overarching strategies that successfully mitigated trade disruptions, as well as frequent challenges across varied geopolitical and economic contexts. The analytical process involved detailed case descriptions to highlight similarities and differences in GHE impacts and trade response effectiveness. This structured analysis underscored the necessity of incorporating spatiotemporal and subject-specific considerations in formulating trade policies in response to GHEs. This leads to the argument for a nuanced, multidimensional approach to trade recovery policy-making.

### Four GHEs this century

#### The 2009 H1N1 influenza pandemic

The H1N1 influenza emerged in March 2009 in Mexico and the US. On June 11, 2009, the WHO declared it a global public health emergency of international concern [42], with the pandemic alert level peaking on this date [43]. The WHO declared the end of the pandemic in August 2010. In 18 months, it caused more than 18,000 deaths and affected more than 200 countries [44].

#### The 2011 Fukushima nuclear leak

On March 11, 2011, an earthquake struck the Pacific Ocean, causing a tsunami that triggered a nuclear leak [45]. The US announced an import ban on Japanese food from radiation-affected areas. Additionally, South Korea and the European Union issued trade bans, while China, Thailand, and Vietnam required radiation inspection certificates for food produced in Japan.

#### The 2014 Ebola epidemic

In March 2014, the Ebola epidemic broke out in Guinea, Sierra Leone, and Liberia in West Africa. In August 2014, the WHO declared it a GHE. The official report on October 15 revealed 8,997 cases and 4,493 deaths [46]. The WHO announced the end of the epidemic in Sierra Leone, Guinea, and Liberia in November 2015, December 2015, and January 2016, respectively.

#### The 2015 MERS epidemic

In May 2015, the first MERS case was diagnosed in South Korea, with the disease spreading in medical institutions. Thirty-six patients died, and 186 were infected [47]. As the disease did not exhibit sustained human-to-human transmission, it was not classified as an international public health emergency. In December 2015, the WHO declared the end of the outbreak.

### Comparison of the four GHEs with national trade recovery

#### Similarities

1. The H1N1 flu occurred in the wake of the 2008 global financial crisis, further slowing the recovery of the affected countries. Owing to travel restrictions and trade embargoes, the tourism industry lost USD 2.8 billion, with the trade deficit in pork and pork products’ reaching USD 27 million. Mexico’s exports fell by 26% in the first quarter of 2009 [48]. The US economy was struggling and reached a nadir after the subprime crisis. The Dow Jones Industrial Average closed at 6763.29 on March 2, 2009, the lowest since April 1997 [49]. The H1N1 outbreak in April 2009 significantly decreased US GDP, retail sales, and exports of pork and pork products.
2. As the Japanese government could not provide on-time tests for all trade partners, Japan’s agricultural products and food exports to these countries stagnated. In the first quarter of 2011, Japan’s economy contracted at an annual rate of 3.7% [50]. In the aftermath of the earthquake, tsunami, and nuclear leakage, the economy continued to shrink over the next 6 months (GDP fell 0.9% from January to March), and private consumption fell by 0.6%. In September 2012, the government announced that the country was entering a recession [50].
3. The Ebola epidemic affected transportation, tourism, agriculture, and mining. Trading countries and airlines issued travel restrictions to affected areas [46]. Agricultural production was affected, with the epidemic limiting the transport of agricultural products to consumer areas, raising product prices. Conakry’s governor banned Eid celebrations on October 2, 2014 [51]. Travel bans implemented by national authorities and airline flight suspensions [46] cut off trade among West African countries and their partners for about 6 months until August 31, 2014. The loss of workers and travel restrictions reduced mining activity. The US government sent USD 2.89 billion in foreign aid to West Africa, focusing its efforts on Liberia [51].
4. The MERS outbreak reduced the number of tourists visiting South Korea by 2.1 million, resulting in a loss of USD 2.6 billion in tourism revenue. Additionally, the accommodation, catering service, and transportation sectors suffered losses of USD 542 million, USD 359 million, and USD 106 million, respectively [52]. This pushed the transportation sector’s service index below the expected levels in June 2015 and the accommodation and catering industries’ service indexes below the expected levels in June and July 2015.

### Heterogeneities

#### Causes

H1N1 flu was a pandemic caused by viral variants. The Fukushima event was a technological disaster triggered by a strong earthquake but mainly caused by industrialization [53]. The Ebola virus was a highly infectious and destructive disease; the widespread nature of the West African outbreak relates to the highly mobile communities and densely populated regions affected in the early stages [51]. South Korean cases of the MERS virus, which originated in Saudi Arabia, were introduced through international travel.

#### Duration and influence areas

The H1N1 flu lasted approximately 1 year, affecting Mexico and the US. Following the nuclear accident, some countries prohibited agri-food product imports from Japan’s irradiated areas from 2011 to the present (e.g., the US and China).^5^ The Ebola epidemic lasted 2 years, primarily affecting African countries. More than 13,000 confirmed cases were reported globally, with 4,951 deaths and a 36% mortality rate by October 2014. Although the outbreak involved only three countries, there was widespread and intense transmission in the West African region, and four nations (Nigeria, Senegal, Spain, and the US) reported initial cases or localized transmission. The MERS epidemic was challenging for South Korea’s medical system for more than 7 months.

#### Event outcomes

After the H1N1 outbreak, countries restricted travel and banned the imports of pork products, which affected their trade with Mexico, the US, and the rest of the global economy. Unwarranted concerns based on inappropriate designations also led to official and unofficial bans by 17 countries on US pork and pork product imports, with China maintaining its ban until mid-December 2009 [54]. The Fukushima nuclear accident primarily affected Japan’s agricultural product exports because its trade partners were concerned about radioactive contamination [55], while the Ebola epidemic endangered Guinea, Sierra Leone, and Liberia’s economic growth, leading to trade stagnation, foreign investment withdrawal, and a food crisis. MERS negatively affected South Korea’s tourism industry.

Evidence for these event outcomes is as follows.

#### Impact of GHEs on export volumes

**Fig. 2:**
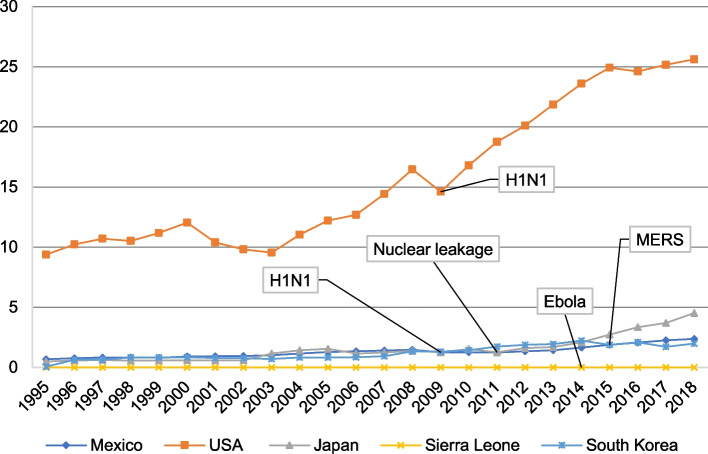
Trends in international tourism revenue changes in GHEs-affected countries, 1995–2018 (current price, USD 10 billion). Data for Guinea and Liberia are missing from the World Bank Database. Source: Author’s analysis based on data from the World Bank Database

In 2009, Mexico and US export volumes decreased by 21.13% and 17.97%, respectively, over the previous year (Fig. 2). Japan’s commodity export volume increased by 6.94% in 2011 over 2010, with a limited share of the Fukushima agricultural food export in Japan’s total foreign trade. Guinea’s commodity exports increased by 10% in 2014 over 2013 but decreased by 13.79% in 2015 over 2014, indicating the Ebola epidemic’s lagging effect on Guinea’s exports. In 2014, Liberia’s and Sierra Leone’s merchandise exports decreased by 54.7% and 19.04%, respectively, over 2013. South Korea’s merchandise exports decreased by 8.02% in 2015 over 2014.

#### International tourism income changes in countries affected by GHEs

**Fig. 3:**
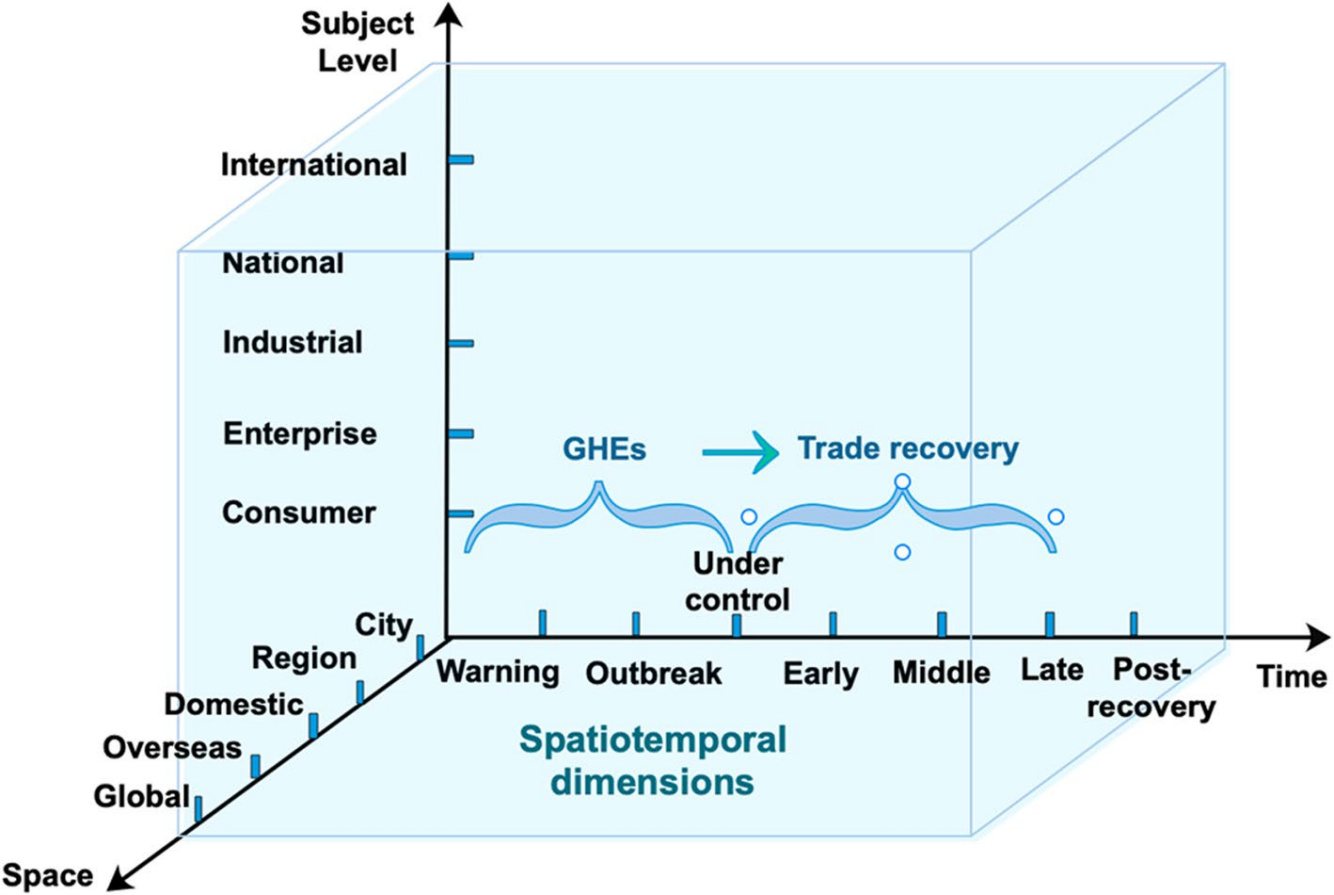
Theoretical framework for the design of trade recovery measures for GHE-affected countries. Source: Author’s analysis

Mexican and US revenues decreased by 14.83% and 11.36%, respectively, in 2009 over 2008, and Japan’s revenues decreased by 18.38% in 2012 over 2011. In Sierra Leone, revenues decreased by 46.97% in 2014 over 2013, and in South Korea, by 16.43% in 2015 over 2014 (Fig. 3). Income from trade and transport fell because of the closure policy adopted during the Ebola outbreak, which also disrupted other business activities [56].

#### Trade recovery

Developed countries (i.e., the US, Canada, and South Korea) have relatively robust health systems, sound economic foundations, and short trade recovery periods. The H1N1 epidemic lasted a year, after which exports from Mexico and the US returned to pre-pandemic levels. As an emerging economy, Mexico maintained its trade with the US during the outbreak; thus, trade recovered rapidly. In 2010, Mexico and US tourism revenues exceeded the level achieved in the 2009 pandemic year.

The impact of the Fukushima nuclear leakage on Japan’s export trade and tourism industry was limited, especially within Fukushima prefecture. Although Japan’s international tourism revenue declined in early 2011, the number of foreign tourists to Japan returned to 70% of that year by September 2012. By contrast, it took more than a decade to eliminate the consequences of the Fukushima disaster on the agricultural product trade. Agriculture production and trade resumed when decontamination was confirmed, which took a long time. In 2017, trade levels improved, and international tourism numbers recovered, exceeding pre-GHE levels [57].

Even before the Ebola outbreak, West African countries were impoverished and pursuing economic development. Guinea, Sierra Leone, and Liberia recovered their export levels within 2 years of the outbreak, but the economic recovery time was long. Guinea’s export recovery was notable; its export trade increased in 2015 over 2014 before decreasing in 2016, although it remained above the pre-outbreak level. In 2015, Liberia’s export volume decreased by 20.57% and did not return to its pre-epidemic level until 2018. Sierra Leone’s exports declined slowly from 2015 until they increased in 2018; however, these are yet to achieve their pre-epidemic level. International tourism income increased by USD 2 million in 2015 over 2014 before fluctuating upward (Figs. 1 and 2).

### Comparison of trade recovery measures in GHE-affected countries

#### Similarities

The common points of the affected countries’ trade recovery measures include countries that chose active fiscal and monetary policies to achieve trade recovery. Consumers, enterprises, and significantly damaged industries were crucial areas for trade recovery.

### Mexico and the US

In May 2009, Mexico implemented a tax rate reduction and funding aimed at small and midsize enterprises in the tourism and transportation industries. Furthermore, it reduced its interbank interest rate and announced a financing plan to inject funds into the economy through institutions (i.e., the National Financial Development Bank) to support small and medium enterprises. The Mexican government revived its economy by introducing rules/regulations to facilitate mergers and acquisitions that promoted the development of the southeast economy within Mexico.

During the H1N1 outbreak, the US economy faced a slowdown in an unstable policy environment following the 2008 financial crisis. The country passed a law to support economic recovery and encourage reinvestment. The Federal Reserve cut interest rates to save financial institutions and enterprises on the brink of bankruptcy and help families with excessive debt. On July 12, 2009, the U.S. Department of Health announced the allocation of an additional USD 1 billion to fight A H1N1 influenza.^6^ Despite reductions in US–Mexico air routes, trade between the two countries continued.

### Japan

In March 2011, Japan launched a post-disaster recovery and reconstruction program, and the Reconstruction Agency was established in 2012. The timeframe included the Intensive Reconstruction Period (2011–2015), with USD 250 billion allocated, and the Reconstruction and Revitalization Period (2016–2020), with USD 65 billion. Japan also established a comprehensive environmental monitoring system.^7^ To accelerate the resumption of normal business operations, the government supported the establishment of temporary stores, increased investment in support funds, and repaired damaged buildings.

Special financial support to reduce enterprises’ burden included establishing a Japanese financial company specialized in recovery and loans intended for reconstruction after the earthquake. The interest rate was slashed, and separate loan limits, extended loans, and repayment terms were established. The interest rate was reduced to almost zero for small and midsize enterprises whose office facilities were destroyed during the earthquake, and the government improved its management and financing. Tourism and other affected industries were supported, and entertainment and consumer destinations, such as Tokyo Disneyland, reopened to revitalize the local economy and restore international confidence after the disaster.

### West Africa

Guinea, Liberia, and Sierra Leone introduced short-term response policies to ensure the health systems and economic sectors’ timely recovery. The Guinean government formulated a USD 2-billion post-Ebola recovery plan, with 63% allocated to improving nutrition, health, education, and children’s services and promoting socioeconomic recovery [43]. It emphasized that the disease’s spread was enhanced by poverty and illiteracy, while noting that the epidemic presented an opportunity to strengthen the country’s economic, social, and institutional resilience. Sierra Leone prioritized the implementation of universal health insurance, whereas Liberia focused on improving post-outbreak areas such as health staffing, infrastructure, monitoring, and response.

The outbreak of a large-scale epidemic in Africa attracted attention from the international community. The United Nations, World Bank, IMF, and US launched a series of epidemic prevention and financial support policies to assist the West African countries in combatting the outbreak. These policies included initial funding of USD 200 million from the US National Institutes of Health to foster cooperation between academic institutions in the US, Liberia, and Sierra Leone on virus research, including vaccine development and new testing and treatment methods. The World Bank approved a USD 110 million IDA assistance to help West Africa establish and expand disease surveillance systems [43].

### South Korea

In June 2015, the central bank of South Korea cut its interest rate to 1.5% [58], issued special financing support, and promoted structural reforms in public utilities, finance, education, and labor sectors. The Korean government provided special insurance for visitors to Korea, covering all medical and MERS-related expenses. The government concurrently introduced supportive policies to reduce consumption taxes on automobiles and large household appliances, offering discounts and organizing shopping festivals. Commercial enterprises offered discounts on commodities and services to stimulate domestic demand and launched the Black Friday Shopping Festival. To accommodate the peak summer vacation from late July to early August and absorb the demand for popular products flowing overseas during the epidemic, Korean enterprises actively supported tourism recovery and extended the discount season from winter until August to attract consumers.

### Heterogeneities

#### The main measures of trade recovery

After the GHEs, the affected governments implemented internal countermeasures to recover. Korean commercial enterprises also participated in the recovery process through marketing measures. Conversely, although the three West African countries implemented internal trade recovery measures, given their economic development and medical infrastructure level, they required additional support from the international community to recover.

#### Emphasis on trade recovery

Most countries strengthened entry-exit control and ensured strict isolation to prevent an epidemic. International flights were reduced, with some countries isolated. Mexico and the US, however, maintained trade ties during the H1N1 influenza pandemic. After controlling the pandemic, the countries used fiscal and monetary policies to manage the affected tourism and agricultural trade. The Japanese government’s trade recovery was based on environmental monitoring measures. When the affected region’s government officials pushed for and promoted marketing measures, it mitigated the nuclear accident’s adverse psychological effects on foreign consumers, thereby advancing the recovery of agricultural exports. The three West African countries’ trade recovery measures are nested in a broader socioeconomic promotion plan. Countries with adequate trade recovery considered the epidemic would opportunistically promote domestic economic development and improve medical facilities with the international community’s support. South Korean commercial enterprises focused on stimulating local demand.

#### Trade recovery measures differ between developed and emerging economies

During the MERS outbreak, South Korea implemented an economic stimulus plan to assist domestic enterprises. Developed economies, such as the US and Japan, also developed support measures for small and midsize enterprises. During the Ebola epidemic, however, West African countries could not provide such resources, and international organizations, such as the World Bank, United Nations Children’s Fund, and WHO, came to their rescue (Table 2).

**Table 2:** Comparison of trade recovery measures of the four GHEs

Events	H1N1	Nuclear Leak	MERS	Ebola
**Dimension**				
Time	Delayed government response. In the early stage, the response was slow. Then, comprehensive measures were strengthened. The government, enterprises, institutions, and society were mobilized	Delayed government response. Information was blocked and delayed. Subsequent efforts included decontamination and rebuilding initiatives, aiming to restore public confidence and rehabilitate economies	Delayed government response. Cooperation with affected medical institutions, isolation of people suspected of carrying the virus, and economic relief for enterprises negatively affected by MERS	Delayed government response. The government focused on working with aid organizations to control the outbreak. Establishing health checkpoints and community education programs to prevent further spread and facilitate quicker medical responses
Space	Mexico and the United States are both members of the free trade area, and the latter is the largest trading partner of the former. Their shared border facilitates substantial cross-border trade, requiring coordinated health and trade responses	The nuclear leak affected Japan’s exports. Miyagi and Iwate Prefectures focused on revival. Fukushima Prefecture needed to end the nuclear radiation disaster and revive global confidence in its agricultural products	The main economic damage to South Korea was the loss of tourism. The government curbed the epidemic without international assistance. Efforts to revive tourism included targeted marketing and safety protocols to attract visitors	The three countries in West Africa could not respond effectively to the epidemic. International assistance was imperative. Subsequent initiatives focused on strengthening healthcare infrastructure and community-based interventions
Subject	Fiscal policy, monetary policy, and regional trade policy. Additional measures included liquidity support for small and medium enterprises and support programs to stabilize employment and assist critically impacted sectors	Environmental monitoring measures, post-disaster reconstruction, financial and monetary policy, and export marketing of agricultural trade. Initiatives to certify and promote the safety of exported goods were intensified to rebuild international trust in affected products	Financial and monetary policy and enterprise marketing strategy. Government support was extended to healthcare sectors, ensuring adequate resources to combat the outbreak effectively. Financial incentives were provided to pharmaceutical companies for the distribution of treatments.	Economic rebuilding and transformation policy. Launching agricultural support programs to rejuvenate the local farming sectors heavily affected. Investment was channeled into infrastructure projects for enhancing connectivity and trade recovery
Characteristics of trade recovery measures	Country and trade partners implemented regional measures to maintain trade. Regulatory adjustments streamlined customs procedures and facilitated cross-border commercial activities	Active fiscal and monetary policies; measures to boost confidence in agricultural products in the affected area; and marketing activities to attract Chinese tourists	Active fiscal and monetary policies; enterprises actively carried out marketing activities to attract and stimulate domestic demand	Unable to avoid the epidemic, cooperated with international aid agencies. Trade recovery, economic reconstruction, and transformation occurred comprehensively

Source: Author’s analysis

### Comparing the cases with the theoretical framework

This study enhances the theoretical framework using case study evidence. Combining the theoretical framework in "Results" section , trade recovery measures in the time dimension of these countries require further clarification, especially when the event was under control and during the trade recovery stage. In the time dimension (Fig. 3), after the GHE was under control (especially after the warning and outbreak), the countries embarked on the process of trade recovery (including early, middle, and late stages).

Implementing the foundations of trade recovery can enhance governments’ timely responses to GHEs. Robust trade recovery infrastructures significantly improve response times during health crises [13, 37, 50]. Trade recovery measures differ based on cities, regions, and domestic countries, with urban centers often rebounding more rapidly owing to better resource allocation [59, 60]. International cooperation is critical for trade recovery, especially for emerging countries, as exemplified by the joint efforts during the 2014 Ebola crisis that facilitated regional trade resumption [61]. The trade recovery measures of developed countries are more comprehensive than those of emerging countries, helping to shorten their recovery time, with the OECD highlighting the correlation between recovery measures and reduced economic downtime [62]. Countries can classify and enrich trade recovery measures by applying the time–space-subject three-dimensional framework analyzed earlier and establishing a countermeasure repository (see Table 3 in "Comparative case study on trade recovery measures after GHEs" section) to recover from GHEs

**Table 3:** Countermeasure repository in time, space, and subject dimensions

Time dimension	**Time frame**	**Phased measures**	**Description**
	Early (3–6 months)	Short-term measures against trade suspension	Fiscal, monetary, and trade countermeasures. Increase expenses; reduce taxes and fees
	Medium (6–18 months)	Medium-term measures against trade contraction	Fiscal, monetary, and trade countermeasures. Provide credit support to enterprises and income subsidies to employees; cash distribution
	Later (18–24 months)	Long-term measures for trade restructuring	Fiscal, monetary, and trade countermeasures. Adjust exchange rates and tariffs; reduce or delay payment of taxes or debts, and interest rates
**Space dimension**	**Area focus**	**Regional measures**	**Description**
	Global health event countries	Epidemic area	Watch for a trade ban. Macroeconomic policies; special support countermeasures; maintain trade activities
		Nonepidemic area	Adopt zoning measures. Negotiate signed trade agreements, expand cooperation opportunities, and take the lead in trade resumption
	Trading partner countries	Major trading partners	Resume different types of trade successively. Strengthen cooperation and ensure trade; improve product quality standards and actively respond to TBTs; ensure bilateral freight transport—some commodity trade can be resumed first; timely contract updates to ensure contract renewal and trade negotiation’s normal progression
		Other trading partners	Safeguard trade networks. Timely consultations on trade recovery, especially for countries that have signed regional trade agreements, focusing on the flow of goods and capital, and expanding the market
**Subject dimension**	**Entity level**	**Strategic measures**	**Description**
	International	Avoid friction	Maintain trade with major trading partners; update and promote signed trade agreements; adjust trade policies and respond to TBTs; sign new trade agreements to encourage trade; correspond with the WHO, WTO, IMF, International Development Bank, and other international organizations as well as regional economic alliances to gain international support and strengthen cooperation
	National	Differentiated approach	Implement active fiscal and monetary policies; distinguish epidemic and nonepidemic areas; implement differentiated prevention and control measures for trade recovery; promote trade normalization in different regions successively; combined with the regional economic development plan, promote the connection between national recovery measures and local trade recovery plans; improve the diversification of foreign trade markets and enhance resilience
	Industrial	Subsidy allocation	Implement subsidy policies for key industries affected by GHEs; track the development trend of foreign markets; promote the establishment of industrial alliances in domestic industries, or rely on existing industrial alliances to encourage industry and trade recovery; promote the internationalization of industrial standards, and maintain and expand foreign markets; release domestic and international industry trend reports and the latest market information; implement targeted support and promotion policies
	Enterprise	Business support	Provide financial and credit support to foreign trade enterprises, especially small and midsize enterprises; accurately recover and reduce taxes and fees; maintain employment rate; facilitate foreign market exploration; encourage the signing of foreign trade agreements; improve trading enterprises’ quality of products and services and enhance international competitiveness; consolidate and expand the foreign trade network; digital transformation; launch marketing measures for domestic and foreign consumers to stimulate demand and achieve product and service innovation
	Consumer	Incentive programs	Launch preferential measures to encourage purchase incentives; restore and improve domestic and international consumers’ purchasing confidence and power; offer time-sensitive promotion or discount on essential and high-demand goods to stimulate immediate consumer spending; introduce or increase tax rebates for consumers who invest in domestically-produced goods or services, incentivizing local spending; facilitate low-interest consumer financing options for high-ticket items to ease the upfront financial burden and encourage purchases; provide clear and transparent information about product sourcing and safety measures to reassure consumers and boost their confidence in the market; sell or promote the purchase of gift cards for future use, providing an immediate influx of cash while encouraging future spending

Source: Author’s analysis

Each GHE revealed areas for improvement in trade recovery measures. The responses to the nuclear leakage accident and the H1N1 influenza epidemic suffered from a lack of timely action and resource allocation [53, 59]. Management of the MERS and Ebola outbreaks has been criticized for insufficient coordination and resource deployment [34]. The outbreak of GHEs has exposed the weaknesses in global governance, manifesting in uncoordinated public health and economic systems, and the failure to manage these events to achieve a better balance among health, economic, and trade shocks. This lack of synergy exacerbates the severity of health, economic, and trade shocks during these crises. Establishing joint committees of the WHO, WTO, and potentially other international organizations, such as the International Monetary Fund and United Nations, could provide a comprehensive approach to managing these conflicts. The effectiveness of such collaborative efforts has been documented in the joint WHO–WTO response to the SARS and H1N1 outbreak, which enhanced global preparedness and response capabilities [63]. Such joint committees could create a real-time data repository for cross-border information sharing, outline a tiered protocol for trade actions, manage a dedicated emergency fund, and conduct bi-annual stress tests. This would not only inform member nations’ preparedness for future GHEs as recommended by the WHO, WIPO, and WTO but also renew their commitment to supporting integrated solutions for global health challenges [64].

Moreover, implementing trade recovery measures in countries affected by GHEs will generate short-term impacts on trade and investment with a delayed effect. According to the Center on Budget and Policy Priorities [65], recovery measures typically result in initial disruptions that are offset by longer-term gains in efficiency and market access. During GHEs, the successive implementation of trade recovery measures influences current economic activities; however, these measures have a delayed and long-term impact. UNCTAD [66] revealed that the full benefits of the trade recovery measures from the pandemic were not realized until several years post-crisis, underscoring the need for patient capital and sustained policy support. Improving the effects of trade recovery measures requires evaluating the implementation effects of the affected country’s measures in response to GHEs, as demonstrated by the World Bank’s analysis of response strategies during the 2014–2015 Ebola outbreak. This provides crucial insights into the effectiveness of regional trade policies [61].

## Discussion

The following countermeasure repository clarifying the time–space-subject dimensions is chosen for countries experiencing GHEs (Table 3). Measures in the time dimension are differentiated in the short, medium, and long terms. Measures in the space dimension strengthen the choices of different geographic areas in the various affected levels (the degree to which an area has been affected by GHEs). The subject dimension highlights the heterogeneity of measures at the international, regional, national, industrial, and consumer levels. Countries experiencing a GHE can choose measures from this repository to address their specific needs.

### Early stage of trade recovery

Countries should implement short-term policies with an open, transparent, and timely response. These policies should include the following.

#### Adopting short-term fiscal and monetary policies

Short-term policies were the primary measures employed by all four countries during the early stages of trade recovery. The availability of open and transparent information helps the government evaluate and control the situation. Timely isolation is significant in controlling an epidemic’s spread, thereby reducing infection and mortality rates. Short-term fiscal spending, income reduction, and credit policies (e.g., tax and reduction of property and insurance fees) can target the most impacted industries. The business cycle prefers a moderately loose monetary policy. Short-term policies should minimize the socioeconomic burden of people affected [67]. For emerging countries, countermeasures to reduce the economic burden are essential for mitigating the adverse effects rather than increasing employment and economic output [33].

#### Implementing trade policies to maintain open trade

During a GHE, neighboring countries and major trading partners fear the epidemic spread through trade channels, triggering trade bans and interruptions. Flight controls and border closures affect countries beyond those implementing the measures [46]. During the Ebola outbreak, West African countries closed their borders, disrupting regional trade and threatening the essential supply and livelihood of the host countries [30, 31]. The affected countries and trading partners should keep trade as open as possible to secure an adequate supply of necessities. During the early stages of trade recovery, reducing trade costs (government-imposed trade costs such as tariffs and quotas) can help protect trade and economic openness. At the international, national, and industrial levels, timely trade policies should be implemented to avoid trade bans and actively respond to technical barriers to trade (TBTs) imposed by other countries. At the national level, affected countries must promptly reduce their short-term trade barriers.^8^ The increased trade barriers during the COVID-19 pandemic further destroyed trade (i.e., the global food system) [68].

#### Middle and later stages of trade recovery

Rapid control of spreading diseases or radioactive substances poses challenges and leads to long-term lag effect on national trade recovery. While quantifying total trade losses from epidemics and nuclear radiation remains difficult, prioritizing national trade recovery is essential for normalizing trade. Measures taken during GHEs should be adjusted based on the overall trade recovery progress to prevent trade friction and expedite the normalization of trade and economic policies. The policy package aimed at ensuring timely trade normalization should incorporate the following three elements.

#### Highlighting macroeconomy-tolerant fiscal and monetary policies

The GHEs significantly disrupted total consumer spending during the middle and later periods of trade recovery. Policy interventions to maintain economic growth are therefore preferable. During GHEs, governments must coordinate their efforts to manage working time arrangements and determine the optimal level of public debt based on production technology and disease characteristics to effectively implement fiscal policy [69]. Simultaneously, medium- and long-term structural policies must be launched while establishing epidemic risk assessment tools. Measures include improving monitoring systems and raising public awareness of prevention and control measures. Pharmaceutical companies should be incentivized to develop new antiviral drugs and vaccines and enhance their production capacity.^9^ Measures to increase medical reserves, such as adopting advanced technologies and medical infrastructure, should be pursued.

#### Supporting key industries and enterprises at medium and micro levels

Efforts include implementing targeted policies for industries significantly affected by the GHE to protect the interests of small and midsize enterprises, particularly those engaged in import and export businesses directly affected. Measures should target preferential policies and subsidies for small and midsize enterprises and prevent unemployment. Enterprises should continue to pay wages and facilitate employee benefit claims despite economic uncertainty. Global manufacturers and retailers can improve e-commerce for shopping channels, develop trust and confidence among e-commerce participants, and promote compatibility with international norms [70].

#### Attracting investment

GHEs can reduce or cause a withdrawal of foreign direct investment from affected countries. When the health event is controlled, implementing tax relief can help reduce losses promptly and promote major investing countries’ and trading partners’ investment plans. For example, foreign investment was withdrawn or withheld during West Africa’s Ebola outbreak. Even after the epidemic was under control, the withdrawn foreign capital slowed the economic recovery of the most affected countries. Weak investment was the primary restraint on trade recovery, accounting for approximately 80% of the decline in goods trade between 2012 and 2016 and between 2003 and 2007 [71]. Countries should actively maintain a stable financial system and encourage foreign direct investment inflows during the middle and later recovery periods.

## Conclusions

This study investigated the impact of GHEs and designed countermeasures to address trade recovery based on theoretical and case analysis. The following conclusions are drawn. First, the unexpected and unique nature of GHEs complicates trade recovery. There were differences in the types of GHEs, their transmission times, and diffusion regions across the four health events. Regardless of the home country’s coping strategy or the experience gained from these events, the trade recovery capability of these countries warrants improvement. Second, the trade recovery measures for the four GHEs were heterogeneous in their focus and effectiveness among developed and emerging economies. Fiscal and monetary policies were more commonly used, followed by recovery measures for specific regions and industries. Enterprises must actively stimulate demand (i.e., marketing, e-commerce). Third, trade recovery measures should be implemented from a spatiotemporal perspective, considering specific subject levels. Short-term policies were the primary focus for affected countries during the early stages of trade recovery. Medium- and long-term policies were crucial for ensuring open trade and trade normalization in the middle and late stages.

The results indicate that trade recovery measures should operate in the space–time-subject dimension. Expedient short-term policies should be adopted during the early stages of recovery (i.e., tax relief and trade subsidies) to stabilize the affected economies rapidly. As recovery progresses, medium- and long-term financial, monetary, and trade policies (i.e., bilateral trade agreements and currency stabilization) should be preferred in the middle and later stages to sustain and bolster economic recovery. Designing trade recovery policies at the international, national, industrial, enterprise, and consumer levels should shift from emergency actions to comprehensive, reserve-oriented, and enduring-effects measures. These policies should address needs at different levels, such as permanent trade corridors to facilitate uninterrupted trade flows and consumer loyalty programs in sustaining market demands.

Our study acknowledges the comprehensive WTO trade measures during the COVID-19 pandemic, which documented diverse practices of trade facilitation and restriction across member states. According to the WTO’s report [72] and further detailed trade policy discussions [73], these measures significantly influenced the economic landscape globally, highlighting the need for adaptable tailor-made trade policies to specific country contexts. Building on these findings, we suggest that future trade recovery strategies should leverage both the resilience measures and lessons learned during the pandemic. Specifically, effective temporary trade measures identified by the WTO can serve as models for swift deployment in future global health emergencies, aiming to minimize disruptions to trade flows.

### Limitations and future research

This study proposed that trade recovery countermeasures designed for countries with GHEs should distinguish between spatiotemporal dimensions and specific subject levels. Different trade recovery countermeasures inevitably produce overlapping effects (i.e., fiscal and easy monetary policies can promote trade recovery). However, this study did not fully explore the interactive or cumulative impacts of these overlapping countermeasures, leaving room for determining the most effective policy combinations. Further research is needed on the superimposed effects of trade promotion and combined policies. For example, clarifying these mechanisms requires analyzing the channels and results of various trade recovery countermeasures affecting trade recovery, collecting quarterly, monthly, even daily, and real-time data from countries with GHEs, and applying difference-in-difference, breakpoint regression models, as well as propensity score matching to identify the mechanism and countermeasures’ effects. This approach can provide insight into the overlapping effects of multiple trade recovery policies.

## Data Availability

The data that support the findings of this study are available from the corresponding author upon reasonable request.

## Abbreviations

GHEs: Global health events
MERS: Middle East respiratory syndrome
WHO: World Health Organization
WTO: World Trade Organization
US: United States
AI: Artificial intelligence
TBT: Technical barriers to trade

## Footnotes

1 GHEs affect the long-run evolution of the economy. This study assumes that countries experiencing GHEs will enter a new stage of development and show a long-run economic evolution.

2 The economy evolves endogenously. A GHE always affects/changes the economy, altering its evolutionary path. Therefore, countries affected by GHEs try to resume or recover trade by implementing trade recovery measures.

3 This causation runs both ways.

4 This research focuses on the impact of GHEs on trade; however, since forever, the relationship has run both ways.

5 The US Food and Drug Administration response to the Fukushima Daiichi nuclear power facility incident (May 14, 2023). https://www.fda.gov/news-events/public-health-focus/fda-response-fukushima-daiichi-nuclear-power-facility-incident Scholars also focus on the motivations/interests of the US agricultural community before and after the event—for example, the nuclear event was just an excuse to prohibit imports. This is also an interesting future topic.

6 US $1 billion to fight against influenza A (H1N1), *China News Report*, October 10, 2022. https://news.ifeng.com/c/7fYidCErT3J

7 Efforts toward reconstruction of Tohoku, Reconstruction Agency of Japan, May 14, 2023. https://www.reconstruction.go.jp/english/index.html

8 Facing COVID-19, the affected countries did the opposite. These activities clearly exposed the weakness of the current recovery measures taken by the countries. Enhancing trade barriers are emergency-oriented measures taken by countries facing multiple uncertainties, while reducing trade barriers are reserve-oriented and enduring-effect measures that benefit countries affected by GHEs.

9 Incentivizing pharmaceutical companies is challenging while recognizing their right to make a profit and the public’s opposing rights and interests. Governments need to play a role in avoiding their monopoly on meeting private interests while encouraging their progress, innovation, and social responsibility.
